# Hypnosis efficacy on nicotine addiction: An analysis of EEG microstates and brain oscillation entropy

**DOI:** 10.3934/Neuroscience.2025002

**Published:** 2025-02-21

**Authors:** Mi Zhang, Junjie Ren, Ni Li, Yongyi Li, Linxi Yang, Wenzhuo Wei, Juan Qiu, Xiaochu Zhang, Xiaoming Li

**Affiliations:** 1 School of Mental Health and Psychological Science, Anhui Medical University, Hefei, Anhui, China; 2 Hefei National Laboratory for Physical Sciences at the Microscale, and School of Life Sciences, University of Science and Technology of China, Hefei, Anhui, China; 3 Department of Psychiatry, Chaohu Hospital of Anhui Medical University, Hefei, Anhui, China; 4 Department of Medical Psychology, School of Mental Health and Psychological Science, Anhui Medical University, Hefei, Anhui, China

**Keywords:** nicotine addiction, hypnosis, EEG microstate analysis, theta oscillations

## Abstract

Despite hypnosis showing efficacy in treating nicotine dependence, its neurobiological impacts on a smokers' brain function remain underexplored. Thirty-three smokers underwent electroencephalography (EEG) recording during pre- and post-hypnosis sessions, each 8 minutes long, alongside Tobacco Craving Questionnaire (TCQ) assessments. Four distinct EEG microstate classes (A, B, C, D) were identified. Daily cigarette consumption negatively correlated with the microstate A duration (r = −0.39, P = 0.03). Hypnosis increased the microstate A parameters while decreasing those of microstate B. Reduced microstate B parameters positively correlated with lower TCQ scores (r = 0.46, P = 0.02). Post-hypnosis, there was a decreased variability and sample entropy in low-frequency theta-band signals, indicating a shift towards more ordered theta oscillations. This shift was inversely related to the microstate D parameters and positively correlated with the microstate C occurrences. Dynamic changes in the brain microstates and theta oscillations elucidate the neurological mechanisms underlying hypnotherapy's effectiveness in treating smoking addiction. These findings provide new insights into the mechanisms by which hypnosis influences brain function and offer potential biomarkers for the treatment of smoking addiction, thus deepening our understanding of therapeutic approaches for substance use disorders.

## 1. Introduction

Nicotine addiction, a pervasive public health issue, is a leading cause of severe illnesses such as lung cancer, cardiovascular disease, and emphysema [1]. As the primary source of nicotine exposure for humans, smoking drives nicotine addiction [2]. According to the World Health Organization, over 7 million people die globally each year from smoking-related diseases, with approximately 6 million being smokers and 1 million non-smokers affected by secondhand smoke [3]. A critical component of the addiction process for smokers is cue reactivity, which refers to the specific psychological and physiological reactions that occur when smokers encounter smoking-related cues [4]. Upon investigating the root causes of nicotine addiction among smokers, we found that cue reactivity is fundamental. An exposure to smoking-related cues triggers a series of specific psychological and physiological reactions in smokers, which further intensify their nicotine dependence [4]. Despite being aware of the negative impacts of smoking and expressing a strong desire to quit, most smokers' attempts to quit end in relapse [5].

Hypnotherapy, a method that alters a patient's behavior, emotions, or perceptions by inducing a hypnotic state and providing suggestions, has proven effective in addressing nicotine addiction [6],[7]. Studies have demonstrated that a significant percentage of smokers who underwent hypnotherapy remained smoke-free in the long term [8]. Moreover, clinical practices have validated the efficacy of hypnotherapy for smoking addiction [9]. By utilizing suggestions and cues during the hypnotic state, therapists aim to modify the smoker's thought patterns and behaviors, thereby aiding in overcoming the urge to smoke [10]. Compared to the high relapse rate and side effects associated with medication [11], hypnotherapy is gaining attention as an alternative treatment for smoking cravings [6]. Despite the significant effectiveness of hypnotherapy for many individuals seeking help to quit smoking, the variability in hypnotic susceptibility among individuals can lead to heterogeneity in the treatment outcomes. These differences are closely linked to a variety of factors, including personal psychological traits (such as suggestibility and levels of focus), previous experiences with hypnosis, and cognitive expectations regarding the treatment [12]. Research indicates that individuals with a higher hypnotic susceptibility are more likely to achieve deep relaxation and heightened states of concentration through hypnosis, which may be one reason they benefit more from hypnotherapy [13].

Electroencephalography (EEG), a pivotal neurodiagnostic modality quantifying cerebral bioelectricity, is extensively utilized in the diagnosis of ailments such as epilepsy, traumatic brain injuries, and sleep disorders [14],[15], as well as in probing the intricacies of cognitive functionality and affective states [16]. The cerebral cortex's electrophysiological patterns exhibit quasi-stable configurations which persist for approximately 80 to 120 milliseconds before transitioning into alternate arrangements, which are designated as microstates [17]. These microstates embody the elemental phases of cognitive processing during both spontaneous and externally elicited neural activities [18], with four archetypal microstates (designated A, B, C, and D) recurrently documented in resting-state EEG research across studies, thus demonstrating a striking cross-individual homogeneity [19],[20]. Nonetheless, the microstates' inherent parameters, including their duration, frequency of occurrence, and spatial distribution, are proven to be influenced by a range of neuropsychiatric pathologies, individual personality profiles, and cognitive operations [21]. Simultaneous acquisitions of EEG and functional magnetic resonance imaging (fMRI) have provided compelling evidence that disparate microstate classes correspond to unique neural networks and functional specializations within the brain [22]. In particular, microstate A, an integral component of the default mode network, exhibits a strong affiliation with the neural activity in the frontal, left insular, and occipital cortices during instances of visual stimulation. Microstate B is consistently implicated in the circuitry supporting visual processing, while microstate C is conjectured to participate in intrinsic mental operations, most notably those that involve self-reflection and the processing of significant information [23]. Lastly, microstate D bears a significant relationship to the neural infrastructure responsible for cognitive control and the allocation of attention [16],[24].

The scientific validity and utility of EEG-derived microstates in the realm of neuroscience have been substantiated through a plethora of applications. These include the assessment of age-related modifications in the spatiotemporal synchronization of brain activity [25], as well as the development of diagnostic and screening protocols tailored to specific clinical populations, such as individuals diagnosed with autism spectrum disorder [26] and schizophrenia [27]. Furthermore, the capacity of a microstate analysis to decode the neurobiological consequences of addictive behaviors on cerebral function is gaining increasing recognition within the academic community [28],[29], thereby highlighting its potential as a powerful investigative tool in the study of complex neuropsychiatric phenomena.

In recent years, a growing body of research has delved into the intricate relationship between neural signal complexity and varying levels of consciousness. Sample entropy (SE), a metric that gauges the regularity and unpredictability of signal patterns, has demonstrated substantial promise in the realm of mental health inquiries, with applications ranging from advancing our understanding of schizophrenia [30] and detecting autism susceptibility in infants [31] to evaluating the developmental maturation of neonatal brains [32]. This metric is grounded in the principle that a lower sample entropy value implies a higher signal regularity; conversely, elevated entropy values signify an augmented level of randomness and signal complexity [33].

Previous studies that utilized a cross-frequency oscillation entropy analysis [34] revealed distinctive entropy patterns associated with modified states of consciousness, which was achieved via meditation or pharmaceutical means [35]. Expanding upon our preceding research into hypnotherapy's efficacy in managing smoking urges [36],[37], this investigation delves deeper into the domain of hypnosis. We adopt k-means clustering to discern the paramount topographical features, thereby classifying them as microstate categories, while concurrently integrating entropy evaluations of oscillatory patterns across various EEG frequency bands with spectral analyses.

Our research is centered on three core objectives: i) mapping the topological structure of microstates and elucidating the sub-second dynamics of brain function; ii) conducting an exhaustive examination of power spectral characteristics across multiple frequency bands in conjunction with oscillation entropy to capture variations in the intricacy of EEG signals; and iii) methodically assessing changes in microstate parameters before and after hypnotic intervention, probing their correlations with clinical assessments, while also probing potential relationships with complexity metrics, SE, within discrete frequency bands.

## 2. Materials and methods

### 2.1. Participants

The study initially enrolled 40 participants, all of whom reported a daily consumption of at least 8 cigarettes over no less than 3 years. Data from seven participants were excluded due to excessive artifacts, leaving a final cohort of 33 subjects. Using G*Power, we confirmed that a sample size of 28 was sufficient to detect a medium effect (f = 0.25) at α ≤ 0.05 and 0.8 power. Basic demographic information was collected, and the hypnotic suggestibility was assessed using the Stanford Hypnotic Susceptibility Scale (SHSS: A). The degree of nicotine dependence was assessed via the Fagerström Test for Nicotine Dependence (FTND) [38]. The clinical characteristics of the participants are presented in Supplementary Table 1. The participants completed the Tobacco Craving Questionnaire (TCQ) [39] both before and following each EEG session, with instructions to abstain from smoking for a minimum of 2 hours preceding the experiment. Written informed consent was secured from all participants beforehand. The Human Ethics Committee at the University of Science and Technology of China granted ethical approval for the research protocol (Approval Number: 2016001).

### 2.2. Study design

**Figure 1.**
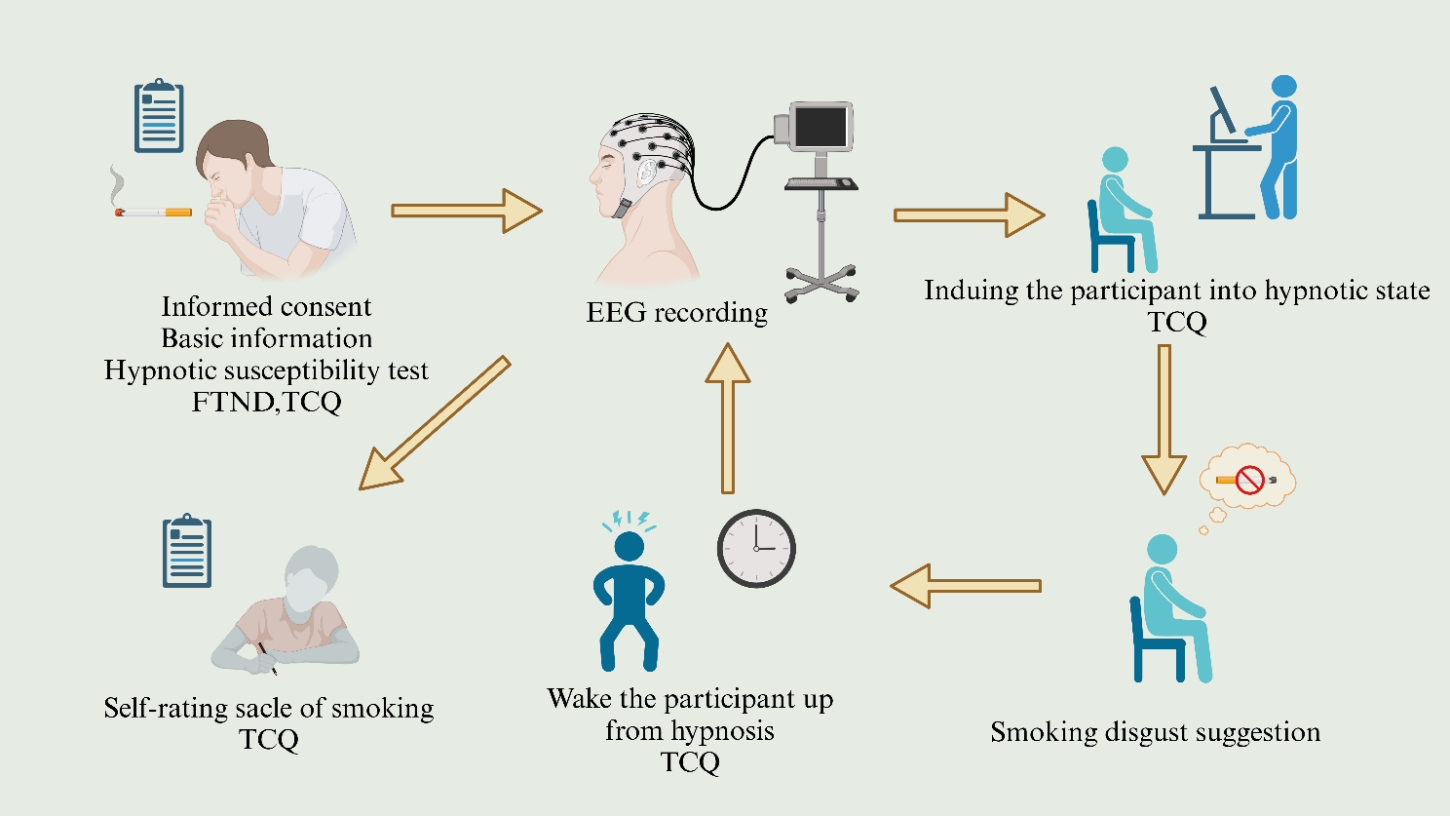
Flowchart of the study. EEG = electroencephalograph; FTND =  Fagerström Test for Nicotine Dependence; TCQ = Tobacco Craving Questionnaire.

This study employed a systematic approach, consisting of two distinct phases: pre-hypnosis and post-hypnotic resting states. Initially, the participants underwent an 8-minute baseline EEG recording while resting with their eyes closed before hypnosis. Subsequently, the participants were then guided through a 15-minute progressive relaxation process designed to help them gradually relax and enter a hypnotic state. Once it was confirmed that the participants had entered the hypnotic state, aversive suggestions were introduced, which were adapted from the research by Spiegel et al. [40]. For instance, the participants were asked to imagine cigarettes emitting a repulsive odor and that smoking would cause extreme discomfort and nausea similar to handling feces. The delivery of these aversive suggestions lasted approximately three minutes. Upon completion of the aversive suggestion phase, the participants were gradually awakened from their hypnotic state through a structured awakening procedure. Finally, in the post-hypnosis phase, EEG recordings were taken again while the participants were in a state of rest to compare with the data collected before hypnosis. A detailed flowchart of the experimental procedure is provided in Figure 1, and the complete script of the aversive hypnotic suggestions is available as Supplementary material Table 2.

### 2.3. EEG acquisition and preprocessing

EEG signals were recorded using a SynAmps2 amplifier (NeuroScan, Charlotte, NC, USA) [41]. A 64-channel Ag/AgCl electrode cap was utilized, with the electrode placement on the scalp following the extended international 10–20 system. The nasal tip of each participant served as the site for the reference electrode, while AFz was designated as the ground electrode to mitigate the potential interference from electromagnetic noise. EEG signals were sampled at a rate of 500 Hz. The participants were guided to maintain a state of wakeful relaxation with their eyes closed, refraining from any active mental engagement.

The preprocessing of raw EEG data was executed utilizing MATLAB, version 2023b. Data were preprocessed using 2 to 20 Hz bandpass filtering. The Infomax independent component analysis (ICA) algorithm was applied to reject artifacts related to eye movement and muscle activity. Finally, the EEG datasets were re-referenced to the average reference.

### 2.4. Microstate analysis

The preprocessed EEG data were imported into the EEGLAB for the microstate analysis. The global field power (GFP) is employed to gauge the intensity of the scalp potentials, with the instantaneous voltage magnitudes at electrodes corresponding to the GFP peak times selected for cluster analysis [42]. GFP signifies the brain's electric field strength at any given moment, typically utilized to quantify the overall response to events or to depict rapid alterations in brain activity, where its peaks denote instances of maximum field strength and an optimal topographical signal-to-noise ratio [24]. Subsequently, to bolster the stability and reliability of the clustering outcomes, a modified K-means algorithm is adopted for iterative computations, set to iterate 100 times. This process ultimately ascertains the optimum number of microstate classes, aligning with the four previously described microstate categories by Koenig—labeled “A”, “B”, “C”, and “D”—which represent distinct patterns of brain activity [19]. Following the microstate identification, pertinent EEG microstate temporal parameters are extracted: duration, occurrence, and coverage. Moreover, we meticulously examined the transition probabilities among the various microstates, with detailed analyses provided in Supplementary Tables 3–5.

### 2.5. Power analysis

Our power spectral analysis concentrated on 2-second segments of noise-free EEG data, sampled at a resolution of 500 Hz. We employed Hanning windows and applied Fast Fourier Transform [43] to assess the activity specifically within the theta (4–8 Hz) and alpha (9–12 Hz) frequency bands. This analytical approach adheres to standard spectral demarcations of brainwave activity and aligns with the frequency domains pertinent to microstate examinations. Given that the Delta (1–4 Hz) and Beta (13–30 Hz) bands extend beyond the core focus of the microstate analysis, they were excluded from our analysis. The relative band power was computed following a standardized protocol, representing the fraction of total power in each specified band relative to the cumulative power across all the analyzed bands.

To comprehensively delineate the dynamic characteristics of the EEG signals, we incorporated the Coefficient of Variation (CV) as a pivotal metric of signal variability. The CV was separately calculated for each frequency band and then aggregated to derive a general measure of the overall EEG variability, thus offering an inclusive insight into the signal's instability profile [44].

### 2.6. Sample Entropy (SE) in different frequency bands

**Figure 2.**
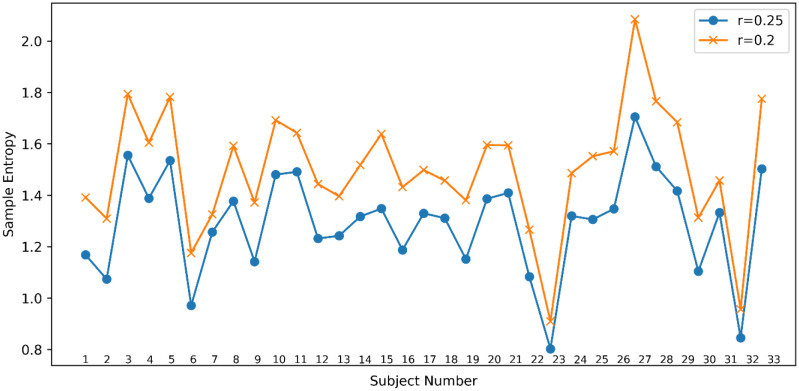
The relationship between tolerance r and sample entropy.

SE functions as a quantitative gauge of the intricacy and unpredictability in time-series data by evaluating the likelihood of repetitive patterns within a defined sequence window, labeled as “m”, and by setting a tolerance threshold, “r”, which discriminates between distinct patterns [33]. As visualized in Figure 2, the results reveal a consistently notable disparity among the participants' EEG signal-derived SE values when the tolerance r is set to 0.25 and 0.2. This observation suggests that the differentiation in EEG signal complexity across individuals at these precise tolerance settings stabilizes at a discernable level. A substantial increase in the SE values is observed as r further decreases, trending towards theoretically infinite values. Thus, “r” = 0.25 was strategically chosen as the benchmark tolerance parameter for our study to optimally delineate the dynamics of signal complexity with heightened precision, while effectively curbing biases potentially introduced by less fitting tolerance selections. The calculation of SE was accomplished using Python version 3.9, thus adopting an embedding dimension of “m” = 2 and a tolerance value of “r” = 0.25 as the operational parameters.

Taking the impact of the signal length on SE stability into account, preliminary validation determined that SE attains a relative stability when the data length N exceeds or equals 1000 sampling points. Thus, N = 1000 sampling points, which is equivalent to a sliding window of 2 seconds, were adopted in this study. The signals were initially filtered through a 4th-order Butterworth band-pass filter to isolate the theta and alpha frequency bands. Subsequently, the Hilbert transform was employed to obtain the analytic form of the signals, thus encompassing an instantaneous amplitude and phase information.

The signals were initially processed through a 4th-order Butterworth band-pass filter to segregate them into theta and alpha frequency bands. Proceeding with this, the Hilbert transform was applied to derive the analytic representation of the signals, thus obtaining both an instantaneous amplitude and phase components. Ultimately, SE was individually computed for each electrode's signal within every frequency band. Then, these values were aggregated to attain a mean SE value per channel, allowing for a systematic evaluation of the sample entropy characterizing oscillatory activities within specific frequency bands [35].

### 2.7. Statistical analyses

All statistical evaluations were conducted using SPSS, Version 27, complemented by data visualization techniques implemented in Python. The research design employed a three-factor repeated-measures analysis of covariance (ANCOVA) to explore the interaction effects between hypnosis intervention (pre-hypnosis and post-awakening stages), the microstate classes (A, B, C, and D), and various parameters (duration, occurrence, and coverage). Four covariates were included to control for potential confounding variables to adjust for their potential interfering impact: the level of hypnotic suggestibility, the daily cigarette consumption, years of smoking, and age. Additionally, paired-sample t-tests were utilized to assess the specific changes in bandwidth complexity and spectral power before and after hypnosis intervention.

In pursuit of a deeper comprehension of the relationship between the intervention efficacy and the individual characteristics, correlation analyses were also performed to ascertain the strength of linear associations between daily cigarette consumption, years of smoking, scores on the FTND, TCQ, SHSS, and the respective parameters of microstate classifications. Furthering our investigation, we conducted exploratory analyses to unravel the intricate interplay between the brain activity's complexity and the EEG microstates' unique characteristics, illuminating potential correlations between SE changes and the specific parameters defining individual microstates.

## 3. Results

### 3.1. Microstate topographies

**Figure 3.**
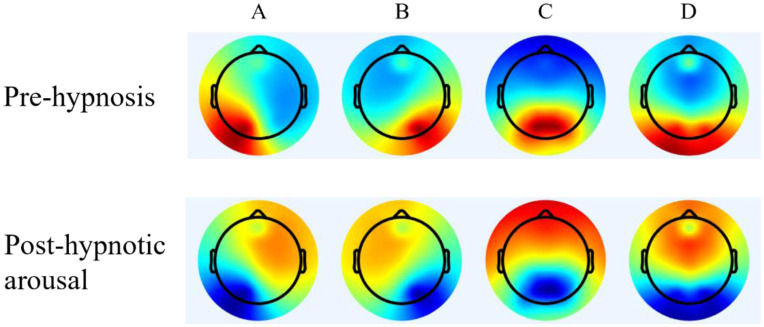
Microstate class topographies.

Figure 3 illustrates the topographic maps depicting the four identified microstate categories, both before and following hypnosis, mirroring those described in prior studies [45]. Our microstate analysis uncovered a quartet of microstate classes: two (labeled A and B) featuring diagonal axis orientations across the topographic map, one (C) with an anterior-posterior alignment, and another (D) characterized by a fronto-central localization. Collectively, these four microstate classes explained over 70% of the total global variance in each group [46], with 70.27% in the pre-hypnosis group and 73.03% in the post-hypnosis group.

### 3.2. Microstate parameters

**Figure 4.**
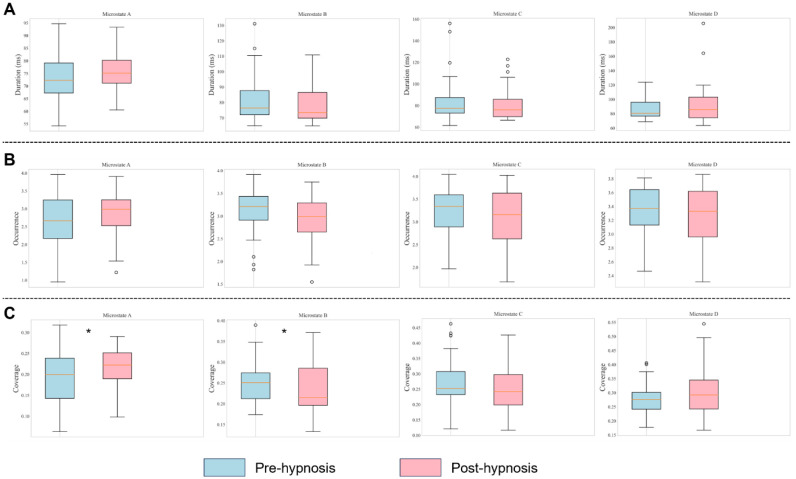
Comparison of microstate parameters between pre-hypnosis and post-hypnosis. A: duration; B: occurrence; and C: coverage.

The study found that the interaction among hypnosis intervention, microstate categories, and various parameters did not reach a statistical significance, F(6, 55) = 1.22, P = 0.31. However, the interaction between the microstates and parameters was significant, F(6, 55) = 3.61, P = 0.004, partial η^2^ = 0.28. A further pairwise comparison analysis revealed that following the hypnosis intervention, the coverage of microstate A significantly increased, P = 0.04, while the coverage of microstate B significantly decreased, P = 0.038. Regarding the remaining microstate categories and their parameters, although not all observed changes reached statistical significance, a trend analysis suggested an upward trend for microstates A and D post-intervention, whereas the parameters for microstates B and C generally showed a declining trend. Detailed data and statistical analyses of these findings are summarized in Table 1 and illustrated in Figure 4.

### 3.3. Spectral analysis and sample entropy

Post-hypnosis, a trend emerged in the EEG activity with an increased power in the alpha frequency band, coupled with a slight decrease in the theta band power. While these shifts hint at directional changes, the statistical analyses failed to establish their significance. Furthermore, no statistically meaningful variations were noted in the CV, a metric of power variability [44], for either of these frequency bands.

Delving deeper into the complexity of oscillations within these frequency bands over time series, a significant decrease in SE was observed in the theta band following the intervention (t = −3.35, P = 0.002). In contrast, the alpha band did not exhibit a comparable statistically significant reduction in SE (t = 1.38, P = 0.18), thus suggesting a more nuanced effect of hypnosis on the complexity of brain oscillations across different frequency domains. Comprehensive details of these findings are presented in Table 2.

**Table 1.** Comparison of the microstate parameters between pre-hypnosis versus post-hypnosis.

	**Pre-hypnosis (Mean)**	**Post-hypnosis (Mean)**	**Pairwise comparison**	
			**d**	**P-adjust**
**Microstate classes A**				
Duration	73.51	75.36	1.85	0.36
Occurrence	2.64	2.85	2.09	0.21
Coverage	0.19	0.22	0.03	0.04*
**Microstate classes B**				
Duration	81.91	78.72	−3.19	0.30
Occurrence	3.10	2.94	−0.16	0.23
Coverage	0.26	0.23	−0.03	0.038*
**Microstate classes C**				
Duration	84.82	81.24	−3.58	0.44
Occurrence	3.20	3.07	−0.13	0.34
Coverage	0.27	0.25	−0.02	0.30
**Microstate classes D**				
Duration	86.49	92.81	6.32	0.44
Occurrence	3.25	3.26	0.01	0.94
Coverage	0.28	0.30	0.02	0.24

Note: * P < 0.05.

**Table 2.** Indices of spectral EEG, coefficient of variation, and sample entropy between pre-hypnosis and post-hypnosis.

	Factor	Spectral indices	Coefficient of variation (CV)	Sample entropy (SE)
Theta	Pre-hypnosis	19.56%	6.02%	1.39
	Post-hypnosis arousal	18.7%	5.42%	1.2
	T-test	1.05	0.79	−3.35
	P-value	0.30	0.44	0.002*
Alpha	Pre-hypnosis	49.69%	13.95%	1.15
	Post-hypnosis arousal	52.76%	12.45%	1.07
	T-test	−1.59	0.10	1.38
	P-value	0.12	0.33	0.18

Note: * P < 0.05.

### 3.4. Correlation analysis

**Figure 5.**
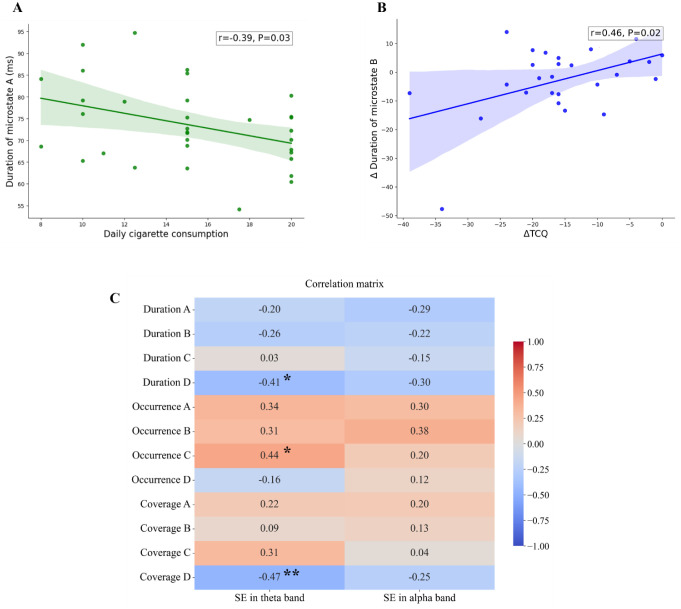
Correlation analysis. **A**: Durations of microstate A and daily cigarette consumption; **B**: Changes in TCQ scores and alterations in the duration of microstate B; **C:** Correlation matrix between SE in theta/alpha band and microstate parameters. A = microstate A; B = microstate B; C = microstate C; D = microstate D; SE = sample entropy; TCQ = Tobacco Craving Questionnaire. ΔTCQ = changes in TCQ scores. *P < 0.05, **P < 0.01.

Our correlation analysis revealed a significantly negative correlation between the daily number of cigarettes smoked and the duration of microstates A (r = −0.39, P = 0.03), a finding visually illustrated in Figure 5A. Moreover, we observed a positive association between alterations in the scores on the TCQ and corresponding changes in the duration of microstates B (r = 0.46, P = 0.02), as demonstrated in Figure 5B.

As illustrated in Figure 5C, in our examination of the relationship between SE and the microstate parameters, we discovered a significant negative correlation between SE in the theta band and the multiple parameters of Microstate D. This finding implies that a decrease in theta oscillations is concurrent with an elevation in the parameters of Microstate D. Furthermore, the theta oscillatory activity shows a positive correlation with the emergence of Microstate C.

## 4. Discussion

This study employed an EEG microstate analysis and brain oscillation entropy to investigate the impact of hypnotic intervention on the dynamic functional properties of the brains of smoking addiction patients. The marked reduction in TCQ scores from pre- to post-treatment supports the positive role of hypnosis in alleviating nicotine dependence (Supplementary Table 6). An initial revelation from our research is the negative correlation between the intensity of smoking behavior, which was quantified by daily cigarette consumption, and the duration of microstates A (r = −0.39). Advancing further, a post-intervention data analysis disclosed significant increments in multiple parameters of microstate A and a declining trend in those of microstate B. Additionally, we noted a substantial post-hypnosis reduction in theta oscillation, which was negatively associated with the parameters of microstate D and positively with the occurrence of microstate C.

Previous research has established that smoking can induce changes in the microstate parameters, such as a reduction in microstate A and an increase in microstate B [29], and that nicotine dependence is associated with widespread anomalies in the brain network connectivity, including alterations in the resting-state functional connectivity within the insular region of young smokers [47], as well as differential effects of short- and long-term nicotine exposure on brain networks [48], and a correlation between heavy smoking and a diminished network efficiency [49]. Our study further refines this framework by meticulously analyzing EEG microstates, thereby uncovering a direct link between smoking addiction and the functionality of specific brain networks, notably those involved in auditory and visual processing.

Our study specifically uncovered a profound influence of hypnotic intervention on the EEG microstates of patients with smoking addiction, thereby demonstrating an increasing trend in the parameters of microstate A following the intervention. This discovery aligns with previous research suggesting that frequent smoking is associated with abbreviated durations of microstate A, thus implying that hypnosis may bolster the operational efficacy of the auditory processing network. Given the established linkage between microstate A and auditory function 23, it is inferred that hypnosis achieves this augmentation by intensifying activity within the auditory network, thereby fostering advanced cognitive processes and potentially reinforcing the commitment and drive to quit. Concurrently, we noted a prominent reduction in the parameters of microstate B after hypnosis, thus suggesting a modulatory suppression of the visual network. Notably, this shift in neural activity patterns coincides with substantial reductions in the subjects' craving scores, providing robust empirical support for our observations. Prior studies have affirmed the critical role of visual cues in provoking smoking cravings [50], and the observation of abnormally heightened activation in the primary and secondary visual cortices among addicted individuals [51] underscores the central role of visual processing regions in addiction mechanisms, thus highlighting their potential as stable biomarkers to assess an addiction predisposition [52],[53]. Therefore, we propose that the hypnosis-mediated attenuation of visual network activity may effectively curtail the brain's responsiveness to visual triggers for smoking, thereby significantly diminishing visually-induced cravings.

Low entropy values signify a high regularity and predictability in data, whereas high entropy reflects disorder and unpredictability [33]. SE, serving as a gauge of time series complexity and forecasting uncertainty about future states, interacts with the brain microstates to reveal profound insights into the dynamic adaptability of information processing and the plasticity of neural architecture, particularly in the theta frequency band crucial for memory, learning, and attention modulation. Within this theta band, we observed a negative correlation between the theta entropy and microstate D, thus indicating that the parameters associated with microstate D—implicated in sustaining focused and vigilant states—are significantly augmented as the theta oscillation increases. This finding not only uncovers the optimized information processing strategies employed by the brain during periods of intense focus and alertness, but also showcases how the brain dynamically reconfigures its functional networks to efficiently allocate cognitive resources in response to environmental demands.

Concurrently, the tight association of microstate C with internal mental processes, such as self-reflection and processing of salient information, underscores its pivotal role in self-cognitive integration [23]. During transitions in conscious states, such as deep sleep or meditation, the enhanced ability of microstate C to balance external stimuli with internal processing suggests a vital part it plays in preserving the continuity of consciousness and psychological stability [54]. The negative correlation between theta oscillation entropy and parameters of microstate C unveils an intriguing mechanism: as the theta oscillations become more ordered, a decrease in microstate C parameters suggests that the brain conserves cognitive resources under a low cognitive load or introspective conditions by simplifying the complexity of information processing, thereby optimizing the in-depth processing of internal information and self-reflection. This interplay highlights the brain's sophisticated tactics in adapting its operational modes to meet varying cognitive demands and maintain mental equilibrium.

Our study has several limitations that deserve attention. First, the absence of a control group may compromise the specificity of evaluating the therapeutic effects of hypnosis intervention. Although a single-group pretest-posttest design holds methodological validity in clinical applications of hypnosis [8],[13], and our previous fMRI studies [37] have confirmed the effectiveness of hypnosis in smoking cessation, eliminating confounding variables remains challenging. To enhance the validity of our findings, we implemented longitudinal multi-time point behavioral data collection and employed multivariate statistical analyses to control for covariates. Nevertheless, establishing clear causal inferences remains problematic. Future research should incorporate a control group that either receives no hypnosis intervention or undergoes non-specific hypnosis to more accurately elucidate the neuro-mechanisms of hypnosis in reducing smoking addiction. Additionally, with a sample predominantly composed of males (only two female participants), the applicability of our findings to nicotine-dependent females is constrained due to underrepresentation [55]. Lastly, our analysis concentrated on the widely researched 2–20 Hz frequency band of EEG microstates, mainly covering activities within the alpha and theta bands. This limitation may narrow our understanding of the brain functionality. Future investigations should broaden their scope to include a full-spectrum analysis, thereby encompassing all bands from delta to gamma, to offer a more comprehensive view of the brain activity and establish a more thorough neurobiological framework for addiction interventions.

## 5. Conclusions

Our study innovatively employed the dynamic shifts in brain microstates and oscillatory entropy within specific EEG frequency bands to unravel the underlying neural mechanisms by which hypnotherapy enhances the treatment outcomes for smoking addiction. Future research should further delve into solidifying the long-term benefits of treatment and refining intervention strategies tailored to different populations, with the ultimate goal of furnishing a more precise and comprehensive approach to the clinical management of nicotine dependence.

## Use of AI tools declaration

The authors declare they have not used Artificial Intelligence (AI) tools in the creation of this article.


