# Supplementary material for: Hypnosis efficacy on nicotine addiction: An analysis of EEG microstates and brain oscillation entropy
Source: AIMS Neurosci. 2025 Feb 21;12(1):15–31. doi: 10.3934/Neuroscience.2025002 (PMC12011983; doi:10.3934/Neuroscience.2025002)
Supplement: Supplementary file 1 [file neurosci-12-01-002-s001.pdf]

AIMS Neuroscience

AIMS Neuroscience, 12(1): 15–31.
DOI: 10.3934/Neuroscience.2025002
Received: 09 November 2024
Revised: 08 February 2025
Accepted: 12 February 2025
Published: 21 February 2025

https://www.aimspress.com/journal/neuroscience

Research article

Hypnosis efficacy on nicotine addiction: An analysis of EEG microstates and brain oscillation entropy

Mi Zhang[1,†], Junjie Ren[1,†], Ni Li[1], Yongyi Li[1], Linxi Yang[1], Wenzhuo Wei[1], Juan Qiu[1], Xiaochu Zhang[2,*] and Xiaoming Li[3,4,*]

[1] School of Mental Health and Psychological Science, Anhui Medical University, Hefei, Anhui, China
[2] Hefei National Laboratory for Physical Sciences at the Microscale, and School of Life Sciences, University of Science and Technology of China, Hefei, Anhui, China
[3] Department of Psychiatry, Chaohu Hospital of Anhui Medical University, Hefei, Anhui, China
[4] Department of Medical Psychology, School of Mental Health and Psychological Science, Anhui Medical University, Hefei, Anhui, China

* **Correspondence:** Email: psyxiaoming@126.com, zxcustc@ustc.edu.cn.

† These two authors contributed equally.

Supplementary

Table 1. Clinical characteristics of the subjects.

Age(year)	25.36 ± 5
Number of cigarettes smoked per day	15.14 ± 3.99
Years of smoking	6.36 ± 3.57
SHSS score	7.52 ± 2.75
FTND score	4.42 ± 2.24

Note: FTND = Fagerström Test for Nicotine Dependence; SHSS= Stanford hypnotic susceptibility scale.

Table 2. Hypnosis aversion suggestion.

Hypnosis aversion suggestion
Now, please imagine in your mind that you are smoking a cigarette. Visualize this cigarette as toxic, emitting an extremely foul odor—an indescribable, unique burnt stench. (Pause for 5 seconds)<br>Now, you can smell this foul odor. Pay close attention to how pungent and nauseating it is. (Pause for 5 seconds) This is a distinct stench that fills you with intense disgust and nausea. Now, fully immerse yourself in experiencing this repulsive smell and the feeling of nausea. (Pause for 10 seconds)<br>Good. Smoking now makes you feel intensely disgusted, nauseous, and uncomfortable. When you awaken from this hypnotic state, you will develop a strong aversion to smoking. Whether you see a cigarette or recall this foul odor, it will make you feel nauseous and dizzy.<br>Each time you consider smoking, this sense of disgust will resurface. You will remember this unbearable stench and the accompanying nausea. Whenever and wherever you think about picking up a cigarette, these sensations will automatically come to mind—powerful and vivid—prompting you to immediately abandon any thought of smoking.<br>Smoking no longer provides you with any satisfaction or relaxation; instead, it only triggers intense discomfort and disgust. Over time, this aversion will grow stronger, ultimately leading you to completely distance yourself from tobacco.<br>Please remain in this hypnotic state and focus on this feeling of disgust and your determination to quit smoking. Allow this feeling to deeply embed itself in your subconscious, becoming an integral part of your life.<br>Good, now please close your eyes and take a moment to rest. During this time, continue to dwell on the feelings you have just experienced, reinforcing this sense of disgust and your resolve to quit smoking.<br>Alright, now slowly open your eyes while staying mindful of everything you have felt. As you fully awaken, this sense of disgust will remain with you and will remind you to stay away from tobacco whenever needed.

Table 3. Table of microstate transition probabilities in nicotine-addicted patients before hypnosis and after hypnotic arousal.

Microstate	Pre to Post	d	T	P (FDR)
	D–A	−0.034	−2.72	**0.008**
Microstate D	D–B	0.01	1.29	**0.000**
	D–C	0.02	1.77	**0.000**
	C–A	−0.04	−3.66	**0.000**
Microstate C	C–B	0.03	2.23	0.354
	C–D	0.00	0.48	**0.000**
	B–A	−0.02	−2.06	**0.000**
Microstate B	B–C	0.03	1.90	**0.031**
	B–D	−0.01	−0.75	**0.020**
	A–B	0.02	1.97	**0.000**
Microstate A	A–C	0.01	0.59	**0.002**
	A–D	−0.02	−1.46	0.214

Table 4. Table of microstate transition probabilities in nicotine-addicted patients after hypnotic arousal.

Microstate	F	P	Comparison	p	d
			D–A vs D–B	0.150	−0.03
Microstate D	4.23	**0.017**	D–A vs D–C	**0.004**	−0.05
			D–B vs D–C	0.148	−0.03
			C–A vs C–B	0.631	−0.01
Microstate C	15.74	**0.000**	C–A vs C–D	**0.000**	−0.08
			C–B vs C–D	**0.000**	−0.07
			B–A vs B–C	0.147	−0.03
Microstate B	8.47	**0.000**	B–A vs B–D	**0.000**	−0.08
			B–C vs B–D	**0.011**	−0.05
			A–B vs A–C	0.367	−0.02
Microstate A	3.38	**0.038**	A–B vs A–D	**0.012**	−0.04
			A–C vs A–D	0.101	−0.03

Table 5. Table of microstate transition probabilities in nicotine-addicted patients before hypnosis.

Microstate	F	P	Comparison	p	d
			D–A vs D–B	**0.000**	−0.07
Microstate D	17.10	**0.000**	D–A vs D–C	**0.000**	−0.11
			D–B vs D–C	0.066	−0.04
			C–A vs C–B	**0.000**	−0.07
Microstate C	25.18	**0.000**	C–A vs C–D	**0.000**	−0.12
			C–B vs C–D	**0.006**	−0.05
			B–A vs B–C	**0.000**	−0.08
Microstate B	11.79	**0.000**	B–A vs B–D	**0.000**	−0.08
			B–C vs B–D	0.743	−0.01
			A–B vs A–C	0.663	−0.01
Microstate A	0.12	0.885	A–B vs A–D	0.677	−0.01
			A–C vs A–D	0.985	0.00

Table 6. Tobacco Craving Questionnaire comparison table.

Comparison groups	TCQ-score (M)	SD	T-test	P-value
Pre-hypnosis vs. Non-suggestion hypnosis	55.39/54.36	12.29/12.66	1.63	0.11
Pre-hypnosis vs. Post-hypnotic aversive suggestion	55.39/42.12	12.29/9.90	6.30	**<0.001**
Pre-hypnosis vs. Post-hypnotic awakening	55.39/46.06	12.29/15.60	3.17	**0.003**

Note: M = Mean; SD = Standard Deviation; TCQ = Tobacco Craving Questionnaire.

AIMS Press

© 2025 the Author(s), licensee AIMS Press. This is an open access article distributed under the terms of the Creative Commons Attribution License (https://creativecommons.org/licenses/by/4.0)
